# Changes in Brain Volumes Are Relevant during Natalizumab-Associated Progressive Multifocal Leukoencephalopathy: Lessons from a Case Report

**DOI:** 10.3390/ijms232113642

**Published:** 2022-11-07

**Authors:** Roberto De Masi, Stefania Orlando, Silvia Armenise, Pantaleo Spagnolo, Ruggero Capra, Maria Carmela Costa

**Affiliations:** 1 Complex Operative Unit of Neurology, “F. Ferrari” Hospital, 73042 Casarano, Italy; 2 Laboratory of Neuroproteomics, Multiple Sclerosis Centre, “F. Ferrari” Hospital, 73042 Casarano, Italy; 3 Complex Operative Unit of Radiology, “F. Ferrari” Hospital, 73042 Casarano, Italy; 4 Multiple Sclerosis Centre, Spedali Civili di Brescia, 25123 Brescia, Italy; 5 Complex Operative Unit of Ophthalmology, “V. Fazzi” Hospital, 73100 Lecce, Italy

**Keywords:** JC virus, multiple sclerosis, progressive multifocal leukoencephalopathy, brain segmentation, MRI post-processing

## Abstract

This is a case report concerning a Natalizumab-associated Progressive Multifocal Leukoencephalopathy (PML) with cerebellar localization and wakefulness disturbances. Awakening and clinical improvement dramatically occurred as soon as the immune reconstitution inflammatory syndrome (IRIS) took place, being it mild in nature and colocalizing with the PML lesion. In these ideal experimental conditions, we applied brain magnetic resonance imaging post-analysis in order to know changes in brain volumes underlying the pathological process over the infection period. White matter volume increased with a decrease in grey matter during IRIS. Conversely, we found a constant increase in cerebrospinal fluid volume throughout the duration of PML, suggesting a widespread abiotrophic effect, far from the lesion. Furthermore, brain parenchymal fraction significantly decreased as expected while the total brain volume remained stable at all times. Neurodegeneration is the main contributor to the steady disability in Natalizumab-associated PML. This process is thought to be widespread and inflammatory in nature as well as sustained by IRIS and humoral factors derived from the PML lesion.

## 1. Introduction

Progressive multifocal leukoencephalopathy (PML) is a rare opportunistic infection caused by the John Cunningham polyomavirus (JC virus or JCV), a widespread agent present latently in the kidneys, urine, and serum of healthy individuals [1]. In conditions of systemic immunodepression such as HIV infection and hematological diseases, JCV can infect and destroy oligodendrocytes, leading to well-known clinically devastating consequences.

This is also the case with immunosuppressive therapies, such as the immune ablative therapy based on Rituximab, which results in a systemic immunosuppression, as well as Natalizumab (NTZ) that induces a cerebral immunosuppression useful in multiple sclerosis (MS) treatment [2]. NTZ-associated PML is the main limitation of this disease modifying therapy (DMT), a monoclonal anti–α4 integrin antibody that selectively inhibits lymphomonocyte migration across the blood–brain barrier (BBB), affecting not only neuroinflammation, but also immune surveillance in the central nervous system (CNS) [3]. Although the bioavailability of NTZ is reduced through drug withdrawal and plasmapheresis, it remains the only possibility to treat PML [4]; the consequent immune restoration is not always beneficial, and a variable proportion of patients with PML worsen due to a severe neuroinflammation, which results from immune reconstitution inflammatory syndrome (IRIS). In particular, PML-IRIS is thought to arise from an excessive protective immune response against pathogen-derived antigens that causes disproportionate tissue damage to the host [4]. Clinical immune reconstitution is commonly associated with neurological worsening and distinct magnetic resonance imaging (MRI) findings that reflect inflammatory changes, including contrast enhancement within the PML lesion (56–87%), oedema (30%), and mass effect (24%) [5]. Consistently, the MRI of PML-IRIS is characterized by the PML lesion associated with inflammatory features that appear on the brain MRI after cessation of an immunosuppressive agent, like NTZ. The inflammatory features, in turn, are constituted by gadolinium enhancement, the main event underlying IRIS. The gadolinium enhancement at onset, before cessation of the immunosuppressive agent, characterizes the inflammatory form of PML (iPML), unlike the common one (cPML), which is completely unenhanced [6], as in the present case.

Other DMTs are also associated with PML, including Fingolimod and Dimethyl Fumarate, but no studies have considered putative changes in brain volumes outside of JCV-induced lesions during the acute phase of infection. This is precisely the topic we have addressed here, in a case of NTZ-induced PML. 

## 2. Case Presentation

Here, we report, after receiving written informed consent subscribed by the patient, the case of a 42-year-old man affected by relapsing remitting multiple sclerosis (RRMS) with disease onset at age 32 with cerebellar ataxia. The diagnosis was supported by the detection of typical MS lesions on a brain MRI and oligoclonal bands (OCB) in the CSF, according to the 2010 McDonald criteria [7] and was followed by beginning therapy. There was no smoking, obesity, or alcoholism, and other inflammatory diseases were also excluded, as there was no comorbidity.

During the ten years of subcutaneously β-interferon (β-IFN) administration three times weekly, the neurological conditions remained stable with the expanded disability status scale (EDSS) score of 1.5. However, due to low therapeutic compliance, β-IFN was suspended in favor of oral 14 mg Teriflunomide mono die administration. 

After about five months, the patient experienced a disabling relapse and a one-point increase in the EDSS, despite a total administration of five megadoses of intravenous (i.v.) 6-methyl prednisolone (6-MP). Specifically, he had a worsening ataxia, going from 1 to 2 on the cerebellar functional score, resulting in an EDSS of 2.5. Considering the enhanced lesion load, we ordered a vertical switching from Teriflunomide to the i.v. Natalizumab therapy every 28-days. Subsequently, the quarterly control visits evidenced a favorable clinical outcome and a normal routine laboratory assessment at the time of the following 56 administrations. These laboratory evaluations included complete blood cell count, plasma electrolytes, and liver and kidney function tests.

The annual MRI of the brain and the cervical spinal cord was also stable. Moreover, due to the ab initio stratify index value of JCV of 0.54, the patient also underwent a surveillance PML protocol. The latter consisted of a fluid-attenuated inversion recovery (FLAIR), diffusion-weighted (DWI), and T1-weighted MRI sequences application every three months. The quarterly evaluation of the stratify index value was always stable, ranging from 0.50 to 0.56 with a mean of 0.52. 

After a long-lasting favorable interval, a mild worsening of the pre-existing ataxia followed the 56th infusion of NTZ. At this point, the patient was admitted to the Complex Operative Unit of Neurology at the “F. Ferrari” Hospital in Casarano (Lecce, Italy) where he was already a patient at the MS Centre. The contextual surveillance MRI (time point 1, T1) evidenced a small faded unenhanced hypointense lesion with hyperintense signal in T1-weighted sequences, suggestive of PML in the left cerebellar hemisphere. Thus, after a lumbar puncture, the polymerase chain reaction (PCR) for JCV on CSF and serum detected 580 and 25 viral copies, respectively, with no abnormalities on routine blood tests. A PML diagnosis was made, NTZ was quickly stopped, and an MRI brain assessment was programmed about every two weeks.

### 2.1. PML Clinical Assessment

From its onset, the infection continued to have a constant clinical core feature, consisting of cerebellar signs of the hemisphere and vermis progressive involvement during the total five months of the acute phase. The temporal sequence of PML symptoms expressed a gradual worsening of the same functional scores at onset without involvement of the novel ones. Therefore, the EDSS score gradually increased from 2.5 at baseline to 9.0 during the hospitalization. 

Specifically, the patient first experienced a mild dynamic ataxia with a subsequent increase in gait impairment to wheelchair use and enticement, which he reached in six weeks. Second, he experienced slurred speech coming from mild to severe dysarthria to anarthria in three weeks; third, the patient experienced dysphagia for solids and then for liquids in two weeks. Finally, he experienced sensory dulling, which required hospitalization in the middle of the third month after the disease onset. In the neurological department, internal medicine complications were treated, such as urinary infections and urinary incontinence, then progressive weight loss and fever until the time of the MRI at T6. At this point, a nuanced hyperintensity was noted at the site of the initial PML lesion and the patient dramatically improved in 24–48 hours. 

This constituted a clinical turning point, since he rapidly recovered normal consciousness, and a gradual neurological improvement began. In fact, there has been a progressive improvement in speech function, and trunk control has taken place. After one month, the repeated lumbar puncture with the absence of viral copies sanctioned the resolution of the infection, and the patient started the neurorehabilitation setting after discharge. Currently, after about a year from the PML, he exhibits an EDSS score of 6.5 with residual blurred speech and walker-use compatible with dynamic ataxia. The recovery from dysphagia and incontinence was complete. In laboratory tests, we never detected alterations as regards inflammation indices, such as the erythrocyte sedimentation rate (ESR), PCR, or fibrinogen, except for their transient increase in conjunction with urinary infection.

### 2.2. MRI Assessment 

The MRI lesion at T1 was defined quickly suspect due to these characteristics: no gadolinium enhancement, involvement of the “U” fibers, hypointense and high hyper-intense signal in T1-weighted sequences, and FLAIR without mass effect.

This lesion, with the same features, underwent a progressive volumetric increase over time, going from about 4 to 25 ml, T1 and T5 respectively, extending also from the cerebellum to the brainstem. This progression was constant up to approximately T5. In fact, T6 evidences an unchanged PML lesion volume compared with the previous one, with a new-onset nuanced hyperintensity within this from early gadolinium enhancement. This was the same pattern we observed in the following MRI from T6 to T8 with the unique difference consisting of the progressive increase in gadolinium enhancement in the edges of the PML lesion.

**Figure 1:**
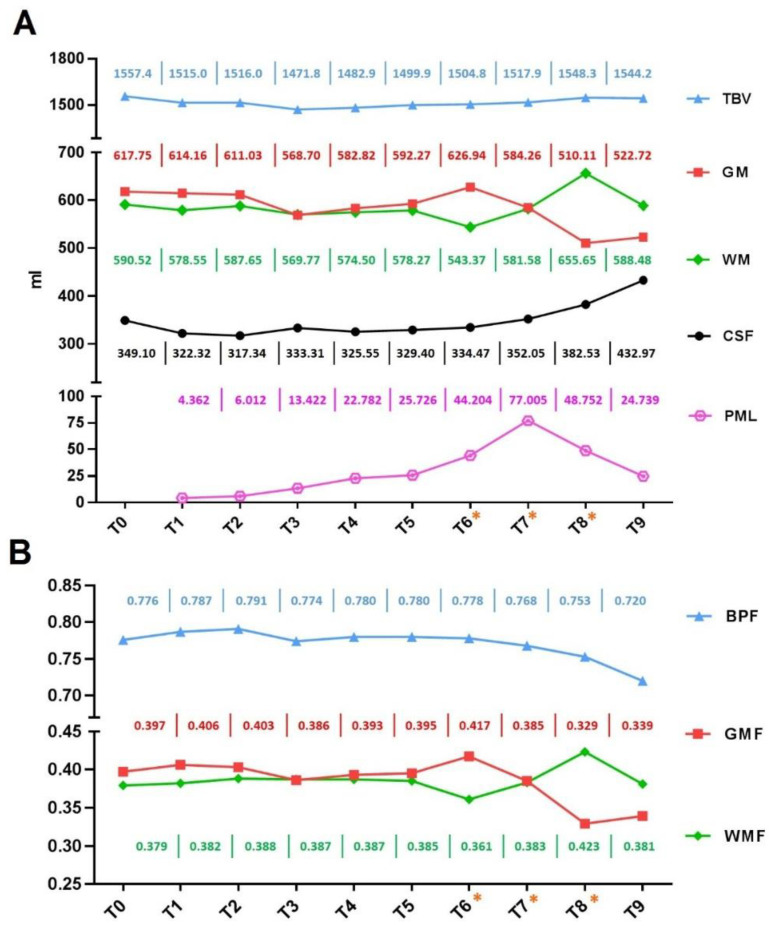
Timeline of changes in brain and PML lesion volumes. Volume (in ml) of total brain (TBV), cerebrospinal fluid (CSF), grey (GM) and white matter (WM), and PML lesion are shown in (**A**); fraction of grey matter (GMF), white matter (WMF), and brain parenchymal (BPF) are shown in (**B**). In both graphs, volumes and fractions curves regarding the entire observation period are represented. Specifically, T0: baseline, T1–T5: PML, T6*–T8*: IRIS, T9: recovery from IRIS. The numerical value of each point is expressed with the same color code as the corresponding curve. PML: progressive multifocal leukoencephalopathy; IRIS: immune reconstitution inflammatory syndrome.

**Figure 2:**
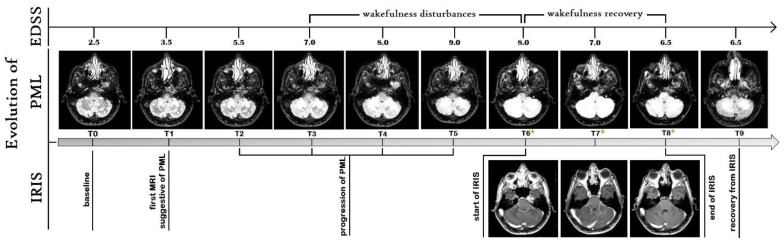
Timeline of evolution of PML, IRIS, and EDSS. Axial brain MRI on FLAIR and T1-weighted post-contrast sequence showing the evolution of PML (top) and IRIS (bottom), respectively. Specifically, T0 shows the baseline MRI, T1 the first MRI suggestive of PML, T2–T5 the progression of PML, T6*–T8* IRIS, and T9 the recovery from IRIS. Note the cerebellar colocalization of PML and IRIS as well as the progressive enlargement of PML lesion from T1 to T5, expressing, the latter, an involvement similar to that of T9, when the infection is over. EDSS is also represented, as well as the time-period characterized by the wakefulness disturbances. Note the progressive accumulation of PML lesion volume and the concomitant worsening of EDSS, from T1 to T5. Wakefulness disturbances manifest in T3 and regress in T6, as soon as IRIS occurs. PML: progressive multifocal leukoencephalopathy; IRIS: immune reconstitution inflammatory syndrome; EDSS: expanded disability status scale.

Furthermore, the T6–T8 period was characterized by an increase in white matter volume (and its fraction, WMF) with the opposite modification in grey matter (and its fraction, GMF). On the contrary, we found a constant increase of CSF, depending on the contribution of periencephalic and ventricular spaces. The BPF decreased as expected while the total brain volume always remained stable. After the end of IRIS (T9), we found a decrease in the volume of grey matter and GMF with respect to baselines. Instead the white matter volume and WMF returned to be comparable to T0 values; finally, the CSF increase and BPF decrease were also confirmed. Figure 1 shows these volumes changes of the brain and PML lesion, while Figure 2 shows the MRI sequences that highlight the evolution of PML and IRIS during the entire observation period. 

In the MRI post-processing, all quantitative measures were calculated by the “Sienax” tool of the FLS package software (created by the Analysis Group, FMRIB, Oxford, UK) according to the voxel-based morphometry methodology. The FSL software is a well-known validated tool for the automatic segmentation of brain volumes and lesion load in demyelinating diseases of the CNS. 

## 3. Discussion

This case report has the merit of describing how brain volumes change during cPML and related PML-IRIS, both exclusively cerebellar. This is the best condition to obtain physiopathological information and understand how JCV- and IRIS-induced inflammation changes brain volumes, as well as their interaction during the acute phase of infection. In fact, our patient manifested cerebellar symptoms, as expected, and wakefulness disturbances. All of these worsened with the enlargement of the iPML lesion but improved as soon as PML-IRIS appeared. From the analysis of clinical data and brain segmentation, interesting findings have been deduced.

First, the JCV-induced lesion, even if only cerebellar, is able to induce a wakefulness disturbance, probably reflecting cytokines diffusion on the cortex and brain hemispheres. This is consistent with the positive effect of PML-IRIS, depending in turn on its cerebellar localization (the same as PML) as well as mild expression, finally affecting viral infection with the improvement of cerebellar but also extra-cerebellar functions, such as wakefulness, precisely. 

Although wakefulness disturbance is an uncommon feature of PML, in our case we think that it is virus-induced in nature due to its rapid resolution in conjunction with the onset of IRIS. Other contributors such as fever and weight loss did not have a temporal relationship with awakening. The latter may have taken place only after the IRIS activity against the virus, resulting in a drastic reduction of widespread inflammation and related humoral factors. In fact, a mild inflammatory activity induced by IRIS is an expected event in the management of PML, often leading to clinical stabilization. This is the reason why we did not administer any corticosteroid therapy. The corticosteroid modulation, on the contrary, is indicated in the case of severe inflammation during IRIS, representing a life-threatening pathology [8].

Second, the IRIS-induced inflammation not only works within infection edges, but also away from them, modifying grey and white matter trophism. Specifically, at the end of IRIS, a decrease of GM volume was found; instead, WM returned to be comparable to baseline values. The latter phenomenon, in part reversible, could be induced by the Wallerian and retrograde degeneration of axons and pyrenophore, secondary to the white matter inflammation [9]. 

Finally, the progressive increase of CSF spaces in the brain, with the concomitant reduction of the parenchymal fraction, does not seem to be limited to the IRIS phase, but involves the whole PML evolution. This means that the widespread neuroinflammation, however induced by cPML and PML-IRIS, results in an overall net effect with consequent inflammatory neurodegeneration. Widespread neuroinflammation is thought to be induced by the humoral diffusion of inflammatory cytokines, deriving first from the PML lesion and then from the IRIS. 

The extent of the resulting neurodegeneration is quantifiable in our patient and it demonstrates an accelerating effect of the abiotrophic phenomenon already detected in MS. In fact, the decrease in BPF from 0.78 to 0.72 is equivalent to that observed over ten years in MS subjects who have an annual rate of 0.5–0.6% in brain volume loss [10].

## 4. Conclusions

Based on our observations, we indicate the inflammatory neurodegeneration as the main physiopathological contributor to the stable disability in NTZ-associated PML. The pathophysiological mechanisms underlying the neurodegeneration in NTZ-associated PML are to be found in the IRIS, which is known to be inflammatory in nature, but also in the PML lesion itself. The latter, in fact, exerts neuroinflammation probably due to the cytokines diffusion, resulting in widespread neurodegeneration and acceleration in the rate of brain volume loss.

## Data Availability

Not applicable.
